# Changing trends of obesity and lipid profiles among Bangkok school children after comprehensive management of the bright and healthy Thai kid project

**DOI:** 10.1186/s12889-022-13712-w

**Published:** 2022-07-09

**Authors:** Chutima Sirikulchayanonta, Vorachai Sirikulchayanonta, Kanjana Suriyaprom, Rachanee Namjuntra

**Affiliations:** 1 Department of Preventive and Social Medicine, College of Medicine, Rangsit University, ฺBangkok, Thailand; 2 Faculty of Science, Rangsit University, Pathumthani, Thailand; 3 Faculty of Medical Technology, Rangsit University, Pathumthani, Thailand; 4 School of Nursing, Rangsit University, Pathumthani, Thailand

**Keywords:** Atherogenic index, Hypercholesterolemia, Hyperlipidemia, Obesity, Schoolchildren

## Abstract

**Background:**

Cardiovascular diseases are the world’s leading cause of death. Children in Thailand are currently facing obesity, hyperlipidemia, and high atherogenic indices. This study aimed to assess the success of the Bright and Healthy Thai Kid project in reducing obesity and high lipid profiles among Bangkok school children.

**Methods:**

A community-based, intervention (participatory action) with pre-post comparison of anthropometric and lipid profile data was conducted in five randomly selected Bangkok primary schools. The participatory action involved teachers, students, and parents. Data collection on anthropometric measurements, dietary intake, physical activity, and fasting blood samples of three generations of students was carried out during July–August (midterm months in Thailand) in the years 2004, 2017, and 2019. SPSS for Windows, version 16 was used for data analysis.

**Results:**

Comparing the data from 2004, 2017, and 2019, obesity rates of 19.3 in 2004 gradually declined to 16.9 and 15.6 in 2017 and 2019 (*P* <  0.001). High serum total cholesterol, triglyceride, LDL-C, and atherogenic indices decreased significantly (*p* <  0.001).

**Conclusions:**

We believe that the great time and effort expended for a campaign to reduce rates of obesity and hyperlipidemia in school children does work to prevent future cardiovascular diseases. Long-term investment in national programs is required to achieve whole societal involvement in improving knowledge and skills related to health, nutrition, and healthy food choices.

**Supplementary Information:**

The online version contains supplementary material available at 10.1186/s12889-022-13712-w.

## Introduction

Cardiovascular diseases (CVDs) are a leading cause of mortality worldwide, taking an estimated 17.9 million people each year [1]. Four-fifths of CVD end of lives are caused by coronary heart disease and cerebrovascular accidents, and one third of them develop in people under the age of 70, including Thailand [1, 2]. Individual risks for CVD are high blood pressure, high blood sugar and lipids, smoking, poor diet, low physical activity, and overnutrition [3]. Rates of overweight and obesity in adults and children continue to grow. From 1975 to 2016, the global prevalence of overnutrition among children and adolescents aged 5–19 years had more than quadrupled, from 4 to 18% [4]. Now, childhood obesity rates in low- and middle-income countries, including Thailand, are rising dramatically. This is a critical problem, and much of its adverse effects on health and society usually last into adulthood. Early detection of the CVD risk factors and appropriate early management from childhood can prevent premature deaths [5]. Our preliminary survey report in 2004 indicated that 40% of the target school-age children had high blood cholesterol [6]. We concluded that their eating habits (high calorie intake, but less fruits and vegetables) and low physical activity were the main factors leading to obesity and dyslipidemia. As we all know, obesity and hyperlipidemia are the main risk factors that lead to atherosclerosis and CVD [7, 8]. A high proportion of Thai children exhibited obesity, hyperlipidemia and high atherogenic indices [6]. Our project “The Bright and Healthy Thai Kid (BAHT) project began in 2004. It aims to improve the nutrition and activities of public-school children by strengthening specific activities with the participation of teachers and families, thus reducing childhood obesity. The purpose of this investigation was to assess the changing trends of obesity and lipid profiles among Bangkok school children after participatory action of the BAHT project.

## Materials & methods

### BAHT project overall research concept and approach

This was a community-based, intervention (participatory action) with pre-post comparison of anthropometric and lipid profile data. Under conceptualization, motivation and technical guidance of our research team, the intervention plan and implementation were participated by teachers, students, and parents.

Our study was actually designed to help the local school systems to solve childhood obesity problems using a holistic participatory approach rather than to test the hypothesis whether an intervention was effective. Participatory action research (PAR) is a recently recognized methodology for intervention, development and change through participation within communities and groups [9, 10]. PAR is appropriate when it is not possible to include a control group for comparison due to the ethical problems. For example, in our research, we would have had to solicit members of a control group (if even possible) that were encouraged to continue with unhealthy lifestyles while also submitting to physical examinations and blood tests which they would perceive as no particular benefit to them. Instead, highly similar schools were selected and all students (not obese students only) were invited to participate in the project. Moreover, the limited budget and resources did not allow us to have a 5-year monitoring system on equal number of similar non-intervention schools without any intervention.

### Intervention strategies

Prior to the intervention, following a general public media campaign, there had been a general public consensus that childhood obesity is a serious problem. This understanding among the key participants was reinforced by the research team before all were ready to go ahead with the intervention. The medium-and long-term goals to minimize obesity rate among the students were set up. Experiences from previous interventions elsewhere were reviewed and discussed. Action plans were then drafted and followed. Monitoring of progress was incorporated into the plan. Routine anthropometric parameters were recorded with yearly monitoring of venous lipid profiles arranged by the research team under consent of the guardians. The data were analyzed and fed back in a timely manner to relevant parties to appropriately modify the succeeding action plan.

### School selection

Inclusion criteria for school selection were that they be public primary schools in the Bangkok Metropolis, under the Office of the Basic Education Commission, Ministry of Education (MOE), and that they be coeducational, with similar demographics for gender, number of students, family socioeconomic status (low to middle class), parental support (including willingness to voluntarily join the project) and with similar school environments. These schools were randomly selected according to the MOE zones; in the first phase (2004–2006), there were two from the inner zone, one from the middle zone and one from the outer zone. After the lag period, in the second phase in 2014, one additional Bangkok public primary school in the inner zone was added because it fit the inclusion criteria and because student obesity rates were quite similar to those of the previous schools that had requested to participate in the study. Therefore, in the second phase, a total of 5 schools, 3 from the inner, 1 from the middle and 1 from the outer area participated the program. Regarding location of the schools, there was a small difference in the playground area among the schools but no difference in accessibility to fast foods, because shopping centers, convenience stores and home delivery services were available everywhere.

### Participants

All our research related to human subjects, human material or human data was handled in accordance with the Declaration of Helsinki. A written informed consent was obtained from both the participants and their parents. Due to ethical problems in humans, we were unable to randomize those who had been tested for blood lipid levels. Participant selection was based on their willingness to participate, and those who declined to participate were respected. However, the school policy was to promote participation. The participating students were in grades 1–6 and 6–12 years old, whereas those who had blood tests were from grades 3 to 6.

With respect to inclusion criteria for students, no students were excluded from general because the interventions were mostly holistically context-based rather than individually based and none of them were physically or psychologically aggressive. Exception included those with serious illness such as heart disease or serious forms of disabilities who were not involved in the physical exercise program. They were also not included in the statistical analysis. All study protocols were assessed and endorsed by the Ethics Review Board, Institute of Research, Rangsit University (Certificate of Approval: COA No. RSUERB 2019–028).

### Data collection

The project was divided into 2 phases; Phase 1: Initial project implementation (2004–2006); Lag period: School self-management (2006–2012); Phase 2: Re-initiation of project (2012–2013; 2014–2019). The new data presented in this study was collected from 2012 to 2019. However, some data from 2004, during the first phase of the BAHT project is also included here for convenient comparison in tables and figures even though it was already published in 2006 [6].

In 2012, baseline surveys including background characteristics, anthropometric measurements, dietary intake and physical activity had been done in the previous 4 participating schools since 2004 to see whether improvements from the BAHT project implementation in the 1st phase still prevailed. After the participatory action intervention was completed (similar to 1st phase) changes in nutritional status were assessed.

In 2014, as mentioned above, one additional school that fit the inclusion criteria voluntarily joined the program, resulting in a total of 5 schools participating in the study.

### Preliminary, intermediate, and post-intervention surveys were conducted in all five schools

Briefly, these surveys included the following activities. For the preliminary survey from 1July to 31 August 2014, we conducted a baseline survey including background characteristics, anthropometric measurements, dietary intake and physical activity of students and parents. For the intermediate survey from 1July to 31August 2016, we followed child anthropometric measurements, dietary intake and physical activity to evaluate progress. For the post-intervention survey from 1July to 31August 2019, we collected data for evaluation of changes in obesity rates and lipid profile including eating and physical activity behavior.

Data collection on anthropometric measurements, dietary intake and physical activity were done annually during July–August (midterm months in Thailand) and in accordance with standard methods (see below). Lipid profiles of school children were taken in the years 2004, 2017, and 2019.

### Anthropometric measurements of weight, height, and waist circumference

All measurements were conducted by two research assistants (BSc. Graduates) who had been trained in exact techniques before data collection. As the support team, they were responsible for data collection from anthropometric measurements, interview questionnaires, data entry and data assessment using the INMU Thai growth program for nutritional assessment (12). They were also assigned to search for recent articles in childhood obesity, to coordinate with teachers and the research team and to prepare for research team meetings. They also helped in notetaking, report writing and some administrative work. The training course for teachers was conducted by the research team augmented with professional guest speakers. Seminars and subsequent visits with parents were conducted by the research team.

Inter-observer variation and intra-observer variation studies were conducted before and during the study period. The two research assistants were standardized by training in the standard techniques to collect weight, height and waist circumference measurements. An inter-rater reliability test was then performed in which the two research assistants were matched with respect to measurements of weight, height and waist circumference of the same group of 10 students. This revealed inter-rater reliability = 1 (100% agreement). Intra-observer variation was checked by repeating the same case measurements for the same outcome.

### Anthropometric measurements

A calibrated digital weighing scale (Seca, Germany) and a calibrated stadiometer (Microtoise) were used to measure weight and height. We used standard methods in Gibson RS (ed) to determine physical parameters [11]. Students wore school uniforms only, without shoes, sweaters and mobile phones and with empty pockets. Weight was recorded in kilogram (with one decimal point), including height and waist circumference in centimeters (with one decimal point). The nutritional status of children was assessed annually as weight for height (WFH) that categorized them into 3 weight groups according to the standards itemized in the Institute of Nutrition Research, Mahidol University (INMU) Thai Growth Program [12]. This program has been used to analyze the nutritional status of Thai children.

Waist circumference was determined during the post-intervention survey in centimeters, 1 decimal point, by using non-elastic tape. It was collected at the navel level as participants stood up and read while exhaling [13]. From the measurements, a waist-to-height ratio (WHtR) was determined. Therefore, a single and unified standard can be identified for all ethnic groups in different populations and in both sexes. A WHtR cutoff value of 0.5 is generally accepted as the international cutoff for pediatric (≥ 6 years of age) and adult central obesity [14, 15].

### Dietary intake & physical activity

Questionnaires for parents consisted of 2 parts. Part 1 was about general characteristics, while part 2 included questions about child dietary intake, frequency questions, physical activity and child rearing practices. The researchers conducted a pilot-testing of the questionnaire with ten parents to ensure that widely consumed foods and typical physical activities were included and entered into the appropriate check-boxes of the food frequency questionnaire (FFQ) section and physical activity questionnaire section (PAQ). The validity of these questionnaires was retested with 30 parents. The FFQ recorded dietary intake and included 26 food items and 9 scale frequencies (from never to 3 times a day. Scores of 1, 2, and 3 were defined as poor, fair and good behavior, respectively, in each category. Details of food analysis have not been included in this report but details were similar to those reported in our previous publication [16].

### Biochemical assessment

Voluntary subjects were informed to fast after 8 PM (10–12 hours) except for small amounts of water. In the morning, collection of 3–5 ml of a fasting venous blood sample from each participant was done by skilled nurses and medical technicians before breakfast. After blood collection, breakfast was offered to the children. Blood samples were sent to a standard, accredited laboratory at the Faculty of Public Health, Mahidol University. We used standard methods to determine the blood lipid parameters [17, 18]. The atherogenic index (AI) was analyzed [6].

### Intervention measures (participatory actions)

In the initial phase, a strategic planning workshop with school directors, teachers, and parents was conducted. To empower teachers to convey the program, a 2-day training course was carried out regarding child health promotion. This included causes and consequences of childhood obesity, information on child nutritional assessment, dietary guidelines, description of healthy school lunches and snacks, guidelines on food safety, and guidelines for exercise and weight management. A total of 120 volunteer teachers from all participating schools and volunteer parents were given lectures and joined small group discussions to gain basic knowledge in health promotion. Their possible roles in obesity problem-solving and engagement in the project were identified. They were encouraged to incorporate what they had learned into their normal daily life with weekly self-reflection and discussion with peers. At school, group physical activities aiming at students’ weight control was carried out every other day. This included aerobic dance for 15 to 20 minutes before the morning class or in the afternoon. Twice a week, healthier lunches with vegetables and fruits (less fried food) were served.

In order to raise awareness, parents and students were given seminars on obesity and its health consequences (including weight control) in each school. Due to the limited size of the meeting rooms, only 100–150 parents / students could attend at a time on a weekend day or weekday at each school. Therefore, 2 to 3 consecutive events were needed to cover the parents / students at each school and a total of 2 months was required to complete this activity. Essay and drawing contests themed “How to be a bright and healthy kid” were organized among the children to inspire them to produce their own ideas on positive behavior to promote health.

Two subsequent visits were made by the research team each year to evaluate the ongoing process and problems encountered in the participating schools. This included guidance and counseling for teachers, parents and students. The nature of parental intervention was to build up child self-discipline in healthy eating, time management and money management and to provide support at home for children with healthy menus including vegetables and fruit, with regular exercise, and with good parental models. Parental compliance with the project objectives was measured or evaluated by their awareness, concern, satisfaction, and responsibility in obesity control. This included subsequent visits of the research team to schools and to meetings with parents where the team could listen to their experiences in building up child self-discipline with respect to diet and physical activity. When some parents encountered problems, other parents who had experience in solving those problems would share their approaches with the group. The research team supported and recorded effective solutions. In addition, the research team asked students in group meetings about their experiences regarding the parental role and support at home.

During the lag phase, there were no incentives provided for the schools. However, the schools, teachers, students and parents still continued to conform to the intervention. Nevertheless, their conformation appeared to fade over time, possibly due to changes in school policies from time based on the interests of the school directors who were often focused more on excellence in student academic performance.

In the second phase (2012–2019), we used the same approach as in the first phase but with additional activities such as signing a memorandum of understanding (MOU) with the schools and gradually increasing additional activities or criteria so that the school could be upgraded from a model school to become a learning center (Details are shown in Table S in Additional file 1).

### Statistical analysis

SPSS (Statistical Package for the Social Sciences) for Windows, version 16 was used for data analysis. Kolmogorov-Smirnov was applied to test normality of the data. Descriptive statistics were utilized to describe the background features of study groups**.** To determine the relationship between variables, the Chi-square test and Pearson’s correlation analysis were used. One-way ANOVA was applied to examine the association between lipid profiles and different age groups including atherogenic indices and years of blood test. Differences were considered to be statistically significant, if the *p*-value was less than 0.05.

## Results

The nutritional status of all school children grades 1–6 was assessed yearly for growth, and percentages of obesity were assessed and recorded. There were 3 generations of students who studied from grades 1 to 6 during the preceding 15 years (2004–2019). In other words, those who finished grade 6 had to leave for a secondary school. An initial survey was conducted in 2004 with a total of 5126 students, and the post-intervention was carried out in the first phase in 2006. After launching the BAHT project (a participatory action project) in 2004–2006, these schools established school policies for healthy school lunches and supportive environments for exercise according to the project concepts. It was demonstrated that the percent obesity gradually declined from 19.3 to 18.0%. In the following tables and graphs, data from the BAHT project from the year 2004 are shown with permission from a previous publication [6] to allow easy comparison with succeeding years.

**Fig. 1:**
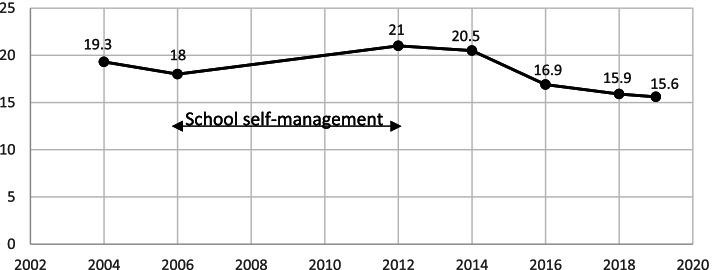
Trends of obesity rates among all primary school children in the BAHT project network during an intervention in the years 2004–2006 and 2012–2019

We had presented the problems of obesity and dyslipidemia among primary school children to the Secretary General of the Office of Basic Education who responded by supporting the National school policy for healthy school lunches and physical activity. There was a lag period from 2006 to 2012 during which we allowed our networked schools to take action in health promotion by themselves, according to their school context, while awaiting the national school policy. We started another nutritional survey in the year 2012 with a total of 4233 students from 4 schools, and it was shown that the obesity rates had risen to 21%. After participatory action intervention has been done (similar to the 1st phase), the obesity rates were slightly diminished to 20.5%. In 2014, a preliminary survey of 5 schools with a total of 5230 students revealed the same obesity rates of 20.5%. After comprehensive intervention (see Intervention measures and Table S in Supplementary file 1) of our project, the percent obesity in our school children gradually decreased from 21% in 2012 to 20.5, 16.9, 15.9, and 15.6% in the years 2014, 2016, 2018, and 2019, respectively, with statistical significance (*P* <  0.001) (Fig. 1).

Regarding blood tests for lipid profiles in the 2019 postintervention survey, there was a total of 725 volunteers of which 47.7% were male, and mean ages were 10.8 ± 0.9 years. The nutritional status was: normal 69.2%, over-weight 28.7%, and underweight 2.1%. The background characteristics of the study groups in 2004, 2017, and 2019 are shown in Table 1.

**Table 1:** Background characteristics of the study groups, who agreed to have their blood tested in 2004, 2017, and 2019

Characteristics	2004*		2017		2019	
	N	%	n	%	n	%
Gender						
Male	535	52.3	344	45.8	346	47.7
female	493	47.7	407	54.2	379	52.3
Age (year)						
Mean ± SD	9.4 ± 1.3		10.1 ± 1.2		10.8 ± 0.9	
6–8	255	24.8	81	10.8	–	–
9–12	773	75.2	670	89.2	725	100
Nutritional status						
Under	31	3.1	27	3.6	15	2.1
Normal	576	56	523	69.6	502	69.2
Over	421	40.9	201	26.8	208	28.7
Total number	1028	100	751	100	725	100

The mean values of lipid levels are indicated in Table 2. In 2019, the mean TC was 185.1± 29.9 mg/dl, while TG was 78.1 ± 36.5, LDL-C was 118.0 ± 27.8 and HDL-C was 52.2 ± 11.2 mg/dl. It was deduced that 38.2 and 29.1%, of the participants had borderline high levels of TC and LDL-C and while 30.5 and 29.1%, of them had high TC and LDL-C. Compared to the preliminary (2004) [6] and intermediate (2017) surveys, it was revealed that the percentages of children with high serum TC, TG, and LDL-C significantly diminished (*p* <  0.001) whereas the percentages with low HDL-C increased in 2017 and gradually decreased in 2019 (*p* <  0.001).

**Table 2:** Lipid levels in 3 generations of students in primary schools that participated in the BAHT project, 2004, 2017, and 2019

Lipid levels (mg/dl)		preliminary	intermediate	Post-intervention	
		2004*	2017	2019	χ^2^
		(*n* = 1028)	(*n* = 718)	(*n* = 725)	*p*-value
		n (%)	n (%)	n (%)	
TC Mean ± SD		194.8 ± 27.7	188.4 ± 31.6	185.1± 29.9	
Desirable	(< 170)	234 (22.8)	196 (27.3)	227 (31.3)	< 0.001
Borderline	(170–199)	391 (38.0)	280 (39.0)	277 (38.2)	
High	(≥200)	403 (39.2)	242 (33.7)	221 (30.5)	
TG Mean ± SD		86.5 ± 31.5	98.0 ± 50.5	78.1 ± 36.5	
Desirable	(< 130)	916 (89.1)	579 (80.6)	663 (91.4)	< 0.001
Borderline	(130–149)	57 (5.5)	56 (7.8)	21 (2.9)	
High	(≥150)	55 (5.4)	83 (11.6)	41 (5.7)	
LDL-C Mean ± SD		125 ± 26.3	116.9 ± 28.0	118.0 ± 27.8	
Desirable	(< 110)	297 (28.9)	295 (41.1)	303 (41.8)	< 0.001
Borderline	(110–129)	315 (30.6)	216 (30.1)	211 (29.1)	
High	(≥130)	416 (40.5)	207 (28.8)	211 (29.1)	
HDL-C Mean ± SD		51.6 ± 8.4	51.9 ± 12.2	52.2 ± 11.2	
Desirable	(≥35)	1017 (98.9)	665 (92.6)	690 (95.2)	< 0.001
Low	(< 35)	11 (1.1)	53 (7.4)	35 (4.8)	

WHtR and lipid levels were significantly correlated, except for LDL-C levels. High TC and HDL-C levels were reported in those with normal WHtR but elevated TG levels were reported more commonly in participants with high WHtR (obesity). There was no relationship between gender and blood lipid levels (Table 3).

**Table 3:** The association between WHtR, gender, and lipid levels in the 2019 study group

Lipid levels (mg/dl)	WHtR			Gender		
	n (%)		χ^2^	n (%)		χ^2^
	≤ 0.5	> 0.5	*P* value	Male	Female	*P* value
TC Mean ± SD	187.2 ± 29.6	180.8 ± 30.4		184.2 ± 29.7	180.8 ± 30.4	
< 170	140 (27.8)	87 (39.2)	0.01	116 (33.5)	111 (29.3)	0.442
170–199	203 (40.4)	74 (33.3)		126 (36.4)	151 (39.8)	
≥ 200	160 (31.8)	61 (27.5)		104 (30.1)	117 (30.9)	
TG Mean ± SD	71.1 ± 31.5	94.2 ± 43.2		76.4 ± 36.8	79.8 ± 37.2	
< 130	477 (94.8)	186 (83.8)	< 0.001	321 (92.8)	342 (90.2)	0.084
130–149	12 (2.4)	9 (4.1)		5 (1.4)	16 (4.2)	
≥ 150	14 (2.8)	27 (12.2)		20 (5.8)	21 (5.5)	
LDL-C Mean ± SD	118.2 ± 27.4	115.9 ± 29.9		117.8 ± 28.1	117.2 ± 27.8	
< 110	204 (40.6)	99 (44.6)	0.454	144 (41.8)	159 (42.0)	0.393
110–129	153 (30.4)	58 (26.1)		108 (31.2)	103 (27.2)	
≥ 130	146 (29.0)	65 (29.3)		94 (27.2)	117 (30.9)	
HDL-C Mean ± SD	55.3 ± 10.9	47.1 ± 10.0		52.2 ± 11.8	53.4 ± 10.9	
< 35	13 (2.6)	22 (9.9)	< 0.001	22 (6.1)	13 (3.4)	0.082
≥ 35	490 (97.4)	200 (90.1)		324 (93.9)	366 (96.6)	

It was revealed that TC had a high correlation with LDL-C (r = 0.894). TC and HDL-C had very low degrees of negative correlation with age; in other words, the more the age, the less the levels of TC and HDL-C (Table 4).

**Table 4:** Correlation coefficients of age and lipid profile of school children, 2019

Variables	TC	TG	HDL-C	LDL-C
Age	−0.087*	0.025	−0.149**	−0.024
TC		0.109**	0.387**	0.894**
TG			−0.399**	0.038
HDL-C				0.111**

* *p* < .05, ** *p* < .01

Mean lipid levels among different age groups in 2019 are shown in Table 5. Atherogenic indices (AI) in the year 2004, 2017, and 2019 significantly decreased as indicated in Table 6.

**Table 5:** Mean lipid levels among 725 students and age groups in 2019

Lipid profile (mg/dl)	Age (years)				F	*P*-value
	9 (*n* = 41)	10 (*n* = 225)	11 (*n* = 268)	12 (*n* = 191)		
TC	196.7 ± 26.0	186.8 ± 31.5	183.5 ± 29.4	183.3 ± 29.5	2.794	0.039
TG	76.6 ± 28.7	77.2 ± 32.7	78.3 ± 39.3	79.6 ± 40.2	0.171	0.916
LDL-C	122.3 ± 23.9	117.6 ± 28.5	116.8 ± 27.6	117.4 ± 29.5	0.451	0.717
HDL-C	59.1 ± 13.3	54.2 ± 11.1	51.6 ± 10.8	51.5 ± 11.4	7.325	< 0.001

**Table 6:** Atherogenic indices of primary school children in 2004, 2017, and 2019

Atherogenic indices	2004 * (*n* = 997)	2017 (*n* = 751)	2019 (*n* = 725)	F	*p*-value
	Mean ± SD	Mean ± SD	Mean ± SD		
TC / HDL-C	3.8 ± 0.7	3.8 ± 1.0	3.6 ± 0.8	14.879	< 0.001
LDL-C / HDL-C	2.5 ± 0.7	2.4 ± 0.8	2.3 ± 0.7	15.798	< 0.001
(TC - HDL-C)/ HDL-C	2.8 ± 0.7	2.8 ± 1.0	2.6 ± 0.8	14.879	< 0.001

According to food frequency questionnaires (FFQ) completed by the parents in 2004, the most popular dish was fried chicken, followed by sausages, and cakes, respectively. More than two-thirds of them favored sweet food. Regarding snacks, the most preference was for potato crisps while fruits and vegetables were often disliked by most children. It was shown that normal-weight children consumed fried chicken less often than obese children (*p* <  0.05) [6]. After the intervention from 2014 to 2019, the new-generation students have consumed more vegetables and fruits due to healthy school lunches with daily vegetables and with fruits 3 times / week, including reduction of fried food. They have also been trained in healthy food choices with more support from parents.

Regarding physical activity, the lower elementary school children spent more time playing, exercising and participating in the program and spent less time with phones or tablets compared to upper level students. The results of this study are quite similar to those of the preliminary study published in 2006 [6].

## Discussion

In 2004, our preliminary study reported a high prevalence of dyslipidemia [6]. The situation may have been due to the influential effect of popular, western, fast-food advertising, availability, and accessibility from either outlets or home delivery services. In addition, television advertisements and online mass media use very attractive presentations or offer special promotions to enhance consumer use. We believe these were the reasons why children had high intakes of fried chicken, sausages and cakes that are high in cholesterol and saturated fatty acids that cause hyperlipidemia. The TC and LDL-C levels in our study group were relatively high when compared to those in other countries [19–21].

We believe that the significant (*p* <  0.001) decrease in percentage of high serum TC, TG and LDL-C (including elevated HDL-C) together with a decline in percent of obesity of our school children, was due to the school-based multicomponent intervention effect of our BAHT project. It motivated students to make healthier food choices and practice more regular exercise [22, 23]. However, compared with younger children, older children had lower HDL- C levels. This could be explained by the fact that the younger group was more active in playing and exercising. The older students, particularly those in grade 6 rarely had time to join our program activities because they spent more time in tutorial hours for the competitive Ordinary National Education Test and the entrance examinations for secondary schools. Moreover, they appeared to be disproportionally devoted to screen time with electronic devices. This behavior correlated with our survey which reported more obesity in them than in younger students [24]. Our findings were supported by other studies that found a positive association between screen time and overweight and obese children [25–27].

In this report, gender and lipid levels in the study group showed no significant association, and this was similar to our previous report [6]. This report reveals that WHtR and lipid levels were significantly correlated except for LDL-C levels. High TC levels were reported in the normal WHtR children, but this might be counteracted by their higher levels of HDL-C, which might be protective. On the other hand, elevated TG levels were reported more commonly in participants with high WHtR (> 0.5) or in abdominally obese participants who might be more at risk of cardiovascular disease in the future. These results were also supported by other studies [26–28]. In addition, our previous study reported that a child’s nutritional status was positively correlated with blood pressure. In other words, the more the weight, the higher the blood pressure (Odds Ratio = 10.60, 95% CI: 3.75–30.00 for HT) [29].

Regarding atherogenic indices (AI) in the years 2004, 2017, and 2019, there were significant decreases in TC and LDL, and an increase in HDL-C as a result of behavior modification. This consisted of improving lifestyles that included healthy eating and regular exercise among school children that joined the program. If they continue with these healthy lifestyles, their CVD risk factors, i.e., obesity, hyperlipidemia, hypertension, and high AI will be very much reduced.

We became concerned during the situation of the COVID – 19 pandemic that students would have to study online at home and that this would lead to less physical activity and more screen time. Parents would need to pay more attention in providing their children with a healthy diet and in acting as good role models for doing regular exercise. It was reported in our previous study [25] that self-discipline among obese children was statistically lower than in normal children in terms of consumption behavior, spending money and time control (*p* <  0.05). Moreover, by adjusted odds ratio (AOR) the ranking of factors related to childhood obesity were money management, poor home surroundings, poor time control, and long screen time, (AOR, 95% CI; 3.1, 1.1–8.2; 3.0, 1.2–7.5; 2.9,1.6–5.4; 2.6, 1.5–4.6), respectively.

It was recommended that parents and teachers cooperate in self-discipline training for children, particularly with regard to consumption behavior, spending money and time control. This should be done in supportive surroundings that would be beneficial for preventing child obesity while simultaneously promoting the development of self-discipline. This has to be a consistent effort lest obesity rates increase during the COVID – 19 pandemic.

We believe that the great time and effort needed for a campaign to reduce rates of obesity in school children does work and that it is worthwhile as an effort to prevent future cardiovascular diseases and to increase the quality of life of children who will be our future adults. Moreover, it will be of significant economic benefit through reduced future national costs for medical care. The lesson learned from the success of our project were: first, to create awareness and instill a cooperative spirit in all stakeholders (parents, teachers and students); second, to promote school advocacy policies for healthy school lunches and for supportive surroundings for doing exercise; third, to activate teachers / working groups to successfully integrate project concepts into daily programs; fourth, to carry out public relations and communication campaigns. Finally, we elicited cooperation from other heath networks (Sweet enough project; Less salt project; Vegan & Fruit 400 g) to promote personal skill development in healthy eating and healthy food choices.

## Strengths of the study

Using participatory action approach, the intervention program was smooth and had a relatively long-lasting effect despite the changes in key actors and participants. Monitoring weight and height including blood lipid profiles, were good evidence-based indicators as to whether there was an improvement in their health as evidenced by a gradual decrease in obesity rates and that coincided with an improvement in blood lipid profiles.

## Limitations of the study

Ethical considerations and the limited budget and resources did not allow us to have a control group. Thus, the changes could also be due to unmeasured confounders and other factors including time trend.

## Conclusions

Childhood obesity diminishes physical, social and mental health. It is generally recognized as one of the risk factors leading to obesity and non-communicable diseases in adults. National policies and extensive response measures are required to generate a healthy environment that can support people in making healthy selections that are dependent on knowledge and skills associated with health, nutrition and healthy food choices. Long-term investment and participation of an entire society to protect children’s rights to good health and well-being are required for sustainability.

## Supplementary Information

**Additional file 1.**

## Data Availability

The datasets used and/or analyzed during the current study are available from the corresponding author on reasonable request.

## Abbreviations

AOR: Adjusted Odds Ratio
ANOVA: Analysis of variance, or ANOVA
BAHT: Bright and Healthy Thai Kid
COVID-19: Coronavirus Disease 2019
CVDs: Cardiovascular diseases
FFQ: Food frequency questionnaires
g: Gram
h: Hour
HDL-C: High density lipoprotein- cholesterol
HT: Hypertension
INMU: Institute of Nutrition Research, Mahidol University
LDL-C: Low density lipoprotein- cholesterol
MOE: Ministry of Education
OPHETS: the Office of Public Health and Environment Technology Services
OR: Odds ratio
PAR: Participatory Action Research
SPSS: Statistical Package for the Social Sciences
TC: Total Cholesterol
TG: Triglyceride
WFH: Weight for height
WHtR: Waist-to-height ratio

## References

[1] World Health Organization. Cardiovascular Diseases. [Cited Nov 12, 2020]. Available from: https://www.who.int/health-topics/cardiovascular-diseases/#tab=tab_1.

[2] Strategy and Planning Division, Office of the Permanent Secretary, Ministry of Public Health. Public Health Statistics A.D.2018. [Cited Dec 12, 2020]. Available from: ps.moph.go.th/new_bps/sites/default/files/statistic 61.pdf.

[3] American Heart Association. Coronary Heart Disease. [Cited Dec 24, 2020] Available from: https://www.heart.org/en/health-topics/consumer-healthcare/what-is-cardiovascular-disease/coronary-artery-disease.

[4] World Health Organization. Obesity. [Cited Nov 12, 2020]. Available from: https://www.who.int/health-topics/obesity#tab=tab_1.

[5] de Ferranti SD, Steinberger J, Ameduri R, Baker A, Gooding H, Kelly AS (2019). Cardiovascular risk reduction in high-risk pediatric patients: a scientific statement from the American Heart Association. Circulation..

[6] Sirikulchayanonta C, Pavadhgul P, Chongsuwat R, Srisorrachata S (2006). A preliminary study of hyperlipidemia in Bangkok school children. Asia-Pacific J Public Health.

[7] Libby P, Buring JE, Badimon L, Hansson GK, Deanfield J, Bittencourt MS (2019). Atherosclerosis Nat Rev Dis Primers.

[8] Mauricio D, Castelblanco E, Alonso N (2020). Cholesterol and inflammation in atherosclerosis: an immune-metabolic hypothesis. Nutrients..

[9] Stringer ET, Stringer ET (2007). Theory and principles of action research. Action research.

[10] The Asia Pacific Observatory on Health Systems and Policies (the APO). Participatory Action Research in health systems. [Cited Mar 25, 2021]. Available from: https://apo.who.int/publications/i/item/participatory-action-research-in-health-systems

[11] Gibson RS, Gibson RS (2005). Anthropometric assessment of body size. Principles of nutritional assessment.

[12] Institute of nutrition, Mahidol University (2002). INMU Thai growth program for nutritional assessment (using weight for height references from national survey, Department of Health, Ministry of Public Health).

[13] McCarthy HD, Jarret KV, Crawley HF (2001). The development of waist circumference percentiles in British children aged 5.0–16.9 y. Eur J Clin Nutr.

[14] Ahmed AY, Watson RR (2019). Comparing Measures of Obesity: Waist Circumference, Waist-Hip, and Waist-Height Ratios. Nutrition in the Prevention and Treatment of Abdominal Obesity.

[15] Yoo EG (2016). Waist-to-height ratio as a screening tool for obesity and cardiometabolic risk. Korean J Pediatr.

[16] Sirikulchayanonta C, Pavadhgul P, Chongsuwat R, Klaewkla J (2011). Participatory action project in reducing childhood obesity in Thai primary school. Asia Pac J Public Health.

[17] American Academy of Pediatrics (1998). Committee on nutrition. Cholesterol Child Pediatr.

[18] The American Academy of Pediatrics*.* Cholesterol Levels in Children and Adolescents. [Cited October 23, 2020]. Available from: https://www.healthychildren.org/English/healthy-living/nutrition/Pages/Cholesterol-Levels-in-Children-and-Adolescents.aspx.

[19] Taheri F, Kazemi T, Bijari B, Namakin K, Zardast M, Chahkandi T (2016). Prevalence of dyslipidemia among elementary school children in Birjand, east of Iran, 2012. J Tehran Heart Cent.

[20] Lartey A, Marquis GS, Aryeetey R, Nti H (2018). Lipid profile and dyslipidemia among school-age children in urban Ghana. BMC Public Health.

[21] Ford ES, Li C, Zhao G, Mokdad AH (2009). Concentrations of low-density lipoprotein cholesterol and total cholesterol among children and adolescents in the United States. Circulation..

[22] Chawla N, Panza A, Sirikulchayanonta C, Kumar R, Taneepanichskul S (2017). Effectiveness of a school-based multicomponent intervention on nutritional status among primary school children in Bangkok. Thailand J Ayub Med Coll Abbottabad.

[23] Mu M, Liu K, He Y (2019). Screen time and childhood overweight/obesity: a systematic review and meta-analysis. Child Care Health Dev.

[24] Laurson KR, Lee JA, Gentile DA, Walsh DA, Eisenmann JC. Concurrent associations between physical activity, screen time, and sleep duration with childhood obesity. Obes. 2014;204540. eCollection 2014. 10.1155/2014/204540. 10.1155/2014/204540 PMC3964685 24734210

[25] Sirikulchayanonta C, Ratanopas W, Temcharoen P, Srisorrachatr S (2011). Self-discipline and obesity in Bangkok school children. BMC Public Health.

[26] Jung MK, Yoo EG (2018). Hypertriglyceridemia in obese children and adolescents. J Obes Metab Syndr.

[27] Elmaoğulları S, Tepe D, Uçaktürk SA, Karaca Kara F, Demirel F (2015). Prevalence of dyslipidemia and associated factors in obese children and adolescents. J Clin Res Pediatr Endocrinol.

[28] Casavalle PL, Lifshitz F, Romano LS, Pandolfo M, Caamaño A, Boyer PM (2014). Prevalence of dyslipidemia and metabolic syndrome risk factor in overweight and obese children. Pediatr Endocrinol Rev.

[29] Sukhonthachit P, Aekplakorn W, Hudthagosol C, Sirikulchayanonta C (2014). The association between obesity and blood pressure in Thai public school children. BMC Public Health.

